# Biomass recalcitrance in barley, wheat and triticale straw: Correlation of biomass quality with classic agronomical traits

**DOI:** 10.1371/journal.pone.0205880

**Published:** 2018-11-07

**Authors:** Francisco J. Ostos Garrido, Fernando Pistón, Leonardo D. Gómez, Simon J. McQueen-Mason

**Affiliations:** 1 Institute for Sustainable Agriculture, Spanish Council for Scientific Research (IAS-CSIC), Alameda del Obispo s/n, Cordoba, Spain; 2 Centre of Novel Agricultural Products (CNAP), Department of Biology, University of York, York, United Kingdom; Tallinn University of Technology, ESTONIA

## Abstract

The global production of cereal straw as an agricultural by-product presents a significant source of biomass, which could be used as feedstock for the production of second generation biofuels by fermentation. The production of sugars for fermentation is an important measure of straw quality and in its suitability for biofuel production. In this paper, we present a characterization of straw digestibility from a wide range of cereal. Our main objective is to evaluate the variability of fermentable sugars released from different species including wheat (*Triticum durum* L., *Triticum aestivum* L.), barley (*Hordeum vulgare* L.) and triticale (X *Triticosecale* Wittmack). To this end, we adapted a saccharification method (IAS Method) capable of detecting significant differences of released sugars between cultivars and species, while using separately another method that would serve as a control and with which we could contrast our results (CNAP method). ANOVA analyses revealed that barley has a higher saccharification potential than wheat and triticale and shows more variation between genotypes. Thus, populations derived from crosses among them such as Steptoe × Morex and OWB Dominant × OWB Recessive hold potential for the identification of genetic basis for saccharification-related traits. The correlation of glucose released between the two methods was moderate (R^2^ = 0.57). An evaluation of the inter- and intra- specific correlation between a number of chemical and agronomical parameters and saccharification suggests that the cell wall thickness and lignin content in straw could be used in breeding programs for the improvement of the saccharification potential. Finally, the lack of correlation between grain yield and saccharification suggests that it would be possible to make a selection of genotypes for dual purpose, low recalcitrance and grain yield.

## Introduction

Widespread burning of fossil fuels produces approximately 81% of the world's energy, of which 41% comes from oil, mostly destined (92%) to the transport sector [1]. The environmental consequences of burning fossil fuels, and the threat of a shortage of energy due to finite oil reserves are well documented [2]. In response, the use of bioethanol as a liquid fuel has triggered a fivefold increase in ethanol production since 2000 [3]. Current commercial biofuel supply relies on first-generation biofuel production, which, although efficient, requires food and feed commodities as a feedstock and as such, poses a potential threat to food security. Although first generation biofuels can be produced efficiently, they use food and feed commodities as a feedstock posing a potential threat to food security. In addition, the cultivation of such feedstocks requires high agrochemical inputs that increase the carbon footprint of biofuels [4]. The development of second-generation biofuels from agricultural waste presents a valuable alternative as it can be obtained as a by-product from food crops [5]. At present, cereal straw is treated as a residue and is usually burnt or incorporated into the soil, but these by-products (including wheat, barley, rice, corn, oat, cotton straw, and bagasse from sugar cane, and totaling approximately 3 billion metric tons annually) present a great potential energy source [6]. Second generation bioethanol production from lignocellulosic biomass requires the conversion of lignocellulose into simple sugars, in three stages [7]: size reduction, thermochemical pretreatment and hydrolysis. The ease with which a biomass is hydrolyzed, also known as saccharification potential, can be used to evaluate recalcitrance of biomass in breeding programs. In this paper, two saccharification methods have been used, one developed by the Instituto de Agricultura Sostenible, (IAS hereinafter) and another procedure, which is widely used, [8] used as a control (Centre for Novel Agricultural Products, CNAP hereinafter).

Pretreatment serves to improve the accessibility of the hydrolysing enzymes to the lignocellulose feedstock. Each pretreatment process is optimised to the biomass to be hydrolyzed since this has a specific effect on the cellulose, hemicellulose and lignin fraction [9]. Due to the great variability in the composition of lignocellulosic materials, it is necessary to adapt the saccharification method to the properties of the biomass. The pretreatment conditions should be chosen in accordance with the configuration of the process selected for the subsequent hydrolysis and fermentation steps. This process, besides being crucial in the conversion of biomass to bioethanol, is considered as the second most expensive after the feedstock cost [9].

The variability in the cell wall degradability of lignocellulosic material can be affected by many factors such as genetic [10,11], morphological [12,13], environmental [14,15], experimental technique for releasing sugars [16], and crop harvesting [17,18]. To fully evaluate all sources of variability, it is advisable to take a multi-phase and multi-environment approach [19] with different experimental methods [16].

The deliberate modification of cell-wall properties is challenging considering the high number of genes involved. Indeed, recent findings in *Arabidopsis thaliana* estimate that 10–15% of plant genes are related to cell-wall biology [20]. This is not surprising since cell walls are essential to plants, contributing to pest and disease resistance and providing mechanical support to plant tissues. Consequently, breeding programs for bioethanol production should aim for a balance between saccharification potential and agronomic performance.

A number of biofuel research initiatives have developed high throughput methods for pretreatment and enzymatic hydrolysis (HTPH) to evaluate the saccharification properties of large collections of germplasm with high potential for the production of second generation biofuels [8,21–23].

The aims of this work are to evaluate the variation in sugar yield from straw obtained from *wheat*, *barley* and *triticale* cultivars under rain-fed environments and to select parental genotypes to develop mapping populations to detect QTL for saccharification.

## Material and methods

### Plant material

Four cereal species were studied: *Hordeum vulgare* L., *Triticum aestivum* L., *Triticum durum* L. and X *Triticosecale* Wittmack (Table 1). Triticale, barley and wheat lines were obtained from either the National Small Grains Collection (NSGC) of the United States Department of Agriculture-Agricultural Research Service (USDA-ARS) (https://www.ars.usda.gov/pacific-west-area/aberdeen-id/small-grains-and-potato-germplasm-research/docs/national-small-grains-collection/) or from the Barley and Wild Plant Resource Center, Okayama University (http://earth.lab.nig.ac.jp). When available, accessions used as parental lines in mapping populations were selected with a dual purpose: Firstly, to allow for the identification of mapping populations suitable for studying the genetic bases of saccharification, and secondly to give a fair representation to the variability available in each species, as parental lines are normally selected to be as divergent as possible.

**Table 1:** Plant material used in this work. More information on the genotypes can be found at https://npgsweb.ars-grin.gov/gringlobal/search.aspx.

Accession name^(a)^	Species	Accession number
Apex**	*H*. *vulgare*	PI600966
Azumamugi***	*H*. *vulgare*	J698
Cebada Capa**	*H*. *vulgare*	PI539113
Clipper**	*H*. *vulgare*	PI349366
Dicktoo**	*H*. *vulgare*	CIho 5529
Franka**	*H*. *vulgare*	PI574293
Franklin**	*H*. *vulgare*	PI373729
Fredrickson**	*H*. *vulgare*	CIho 13647
Golden Promise**	*H*. *vulgare*	PI467829
Igri**	*H*. *vulgare*	PI406263
Kanto Nakate Gold***	*H*. *vulgare*	J518
Ko A**	*H*. *vulgare*	PI383935
L94**	*H*. *vulgare*	CIho 11797
Lina**	*H*. *vulgare*	PI584808
Mokusekko 3**	*H*. *vulgare*	PI420938
Morex**	*H*. *vulgare*	Ciho 15773
OWB dominant**	*H*. *vulgare*	GSHO3450
OWB recessive**	*H*. *vulgare*	GSHO3451
Stander**	*H*. *vulgare*	PI564743
Steptoe**	*H*. *vulgare*	CIho 15229
Vada**	*H*. *vulgare*	PI280422
Anza*	*T*. *aestivum*	NA
Avocet**	*T*. *aestivum*	PI464644
BobWhite*	*T*. *aestivum*	NA
Caledonia**	*T*. *aestivum*	PI610188
Cayuga**	*T*. *aestivum*	PI595848
CIGM90.248**	*T*. *aestivum*	PI610750
Excalibur**	*T*. *aestivum*	PI572701
JAYPEE**	*T*. *aestivum*	PI592760
Kanqueen**	*T*. *aestivum*	PI401539
M6**	*T*. *aestivum*	PI83534
McNeal**	*T*. *aestivum*	PI574642
Opata85**	*T*. *aestivum*	PI591776
OS9A**	*T*. *aestivum*	PI658243
P91193**	*T*. *aestivum*	GSTR 10001
P92201**	*T*. *aestivum*	GSTR 10002
Penawawa**	*T*. *aestivum*	PI495916
Perico*	*T*. *aestivum*	NA
QCB36**	*T*. *aestivum*	PI658244
Renan**	*T*. *aestivum*	PI564569
SS550**	*T*. *aestivum*	GSTR 12501
TAM107-R7**	*T*. *aestivum*	GSTR 11601
Thatcher**	*T*. *aestivum*	CItr 10003
UC1110**	*T*. *aestivum*	GSTR 13501
USG 3209**	*T*. *aestivum*	PI617055
Amadina**	*T*. *durum*	GSTR 12701
Avalon**	*T*. *durum*	PI446910
CO940610**	*T*. *durum*	GSTR 10702
Grandin*5/ND614-A**	*T*. *durum*	GSTR 10401
IDO444**	*T*. *durum*	GSTR 12902
Jupateco 73S**	*T*. *durum*	GSTR 10501
NY18/Clark's Cream 40–1**	*T*. *durum*	GSTR 10402
Rio Blanco**	*T*. *durum*	PI531244
Rugby**	*T*. *durum*	CItr 17284
UC1113 Yr36 Gpc-B1**	*T*. *durum*	PI638741
Weebill 1**	*T*. *durum*	GSTR 10502
Armadillo 130**	*X Triticosecale*	PI583701
Currency**	*X Triticosecale*	PI483066
Drira**	*X Triticosecale*	PI520478
Juanillo 95**	*X Triticosecale*	PI520488
Kramer**	*X Triticosecale*	PI476216
Navojoa**	*X Triticosecale*	PI520421
Rahum**	*X Triticosecale*	PI422269
Wapiti**	*X Triticosecale*	PI511870
Yoreme Tehuacan 75**	*X Triticosecale*	PI519876
Zebra**	*X Triticosecale*	PI429031

^(a)^ Plant material availability

* IAS-CSIC

** USDA-ARS, National Small Grains Germplasm Research Facility, Aberdeen, ID 83210, USA

*** *Barley* and Wild Plant Resource Center. Institute of Plant Science and Resources. Okayama University, Kurashiki, 710–0046, Japan.

### Field trials and sample processing

Three field trials, which were designed in three completely randomized blocks, were conducted in Córdoba **(**37.85981, -4.796895). Each field trial included sixty-six accessions belonging to four different species: barley (*Hordeum vulgare L*.) common wheat (*T*. *aestivum*), durum wheat (*T*. *durum*) and triticale (*Triticosecale*) (Table 1). Each plot consisted of four plants separated by 15cm with an inter-plot distance of 30cm and an inter-furrow distance of 50cm. The straw harvested included leaves and stems; and it was harvested at maturity for each genotype. Samples were chopped using a grinder before processing was performed using a cyclonic mill (Cyclotec 1093, Foss-Tekator) with a 1mm sieve.

### Phenotyping

The all genotypes were scored for: plant height at different stages of growth, total plant biomass, grain yield, biomass yield and stem wall thickness at several internodes. All determinations but plant height was taken at harvest.

### Theoretical ethanol yield calculation

The theoretical ethanol yield was calculated considering the total biomass conversion per surface area unit (ha), according to the National Renewable Energy Laboratory Standards (NREL) [24]. Theoretical ethanol was conducted through the following formula:

f(Glu,Biomass)=Glu×0.511×Biomass×1/1000

Where Glu: Glucose released (*μl* · *mg*^−1^*DW*), Biomass: Theoretical biomass (*Kg* · *ha*^−1^), produced by genotype from the quantity of straw in plots of 0.3 square meters, 0.511: theoretical ethanol yield conversion.

### Saccharification systems

Both systems to determine saccharification were calibrated based on previous knowledge for near optimal hydrothermal pretreatment of straw and optimal enzyme loading [25,26].

### IAS method

Assays to determine saccharification involved three main steps: pretreatment, hydrolysis and sugar detection. The conditions established by *Gomez et al*. [8] and *Santoro et al*. [23] were adapted for sample processing in a single 2 mL tube as used by *Santoro et al* [23]. Briefly, 20 mg of ground straw were loaded into 2 mL screw-cap tubes. A pretreatment solution (6.25 mM) NaOH was used as described by *Santoro et al*. [23] using 1.5 mL of pretreatment solution and incubated at 90°C for 3 h in a water bath, then cooled on ice. Enzymatic hydrolysis was performed using an enzyme cocktail with a 4:1 ratio of Celluclast: Novozyme 188 (Novozymes, Bagsvaerd, Denmark) [8]. Hydrolysis was performed during 20 h with constant shaking, at 50°C in a 0.5 M sodium citrate buffer at pH 4.5. Different enzyme concentrations were assayed to optimize the digestion in a single tube (S1 Fig), the concentration selected as optimal to determine differences between genotypes was 0.05 μL/mg DW. Nine serial dilutions were established from a maximum enzyme concentration of 2 μL/mg of dry weight (DW). The determination of sugars released after hydrolysis was carried out using the glucose oxidase/peroxidase (GOPOD) kit (K-Gluc, Megazyme, Ireland). The assay volumes were reduced to allow the procedure to be performed in 96-well ELISA plates. Glucose determination was performed using 8 μL of the digestion reaction mixture and 240 μL of the GOPOD assay reagent followed by incubation at 50°C during 20 min. The yield of glucose was analyzed using 96 well plates. Absorbance readings were determined at 490 nm in a BioTek ELx800 Absorbance Microplate Reader (BioTek Instruments, Inc.). The adapted protocol used at IAS was validated with the saccharification protocol described by *Gomez et al*. [8].

### CNAP method

96-well plates containing biomass underwent saccharification analysis using a liquid handling platform (Tecan Evo 200; Tecan Group Ltd.) which pretreated the samples with 0.5N NaOH at 90°C for 20 min, followed by enzymatic hydrolysis 50°C for 8 hours. The enzyme cocktail contained commercially available Celluclast and Novozyme 188 (Novozymes A/S, Bagsvaerd, Denmark) at a ratio of 4:1 at an enzyme loading of 22.5 Filter Paper Units (FPU)/g. The reducing sugars released during hydrolysis were determined using a colorimetric assay involving 3-methyl-2-benzothiazolinone hydrozone (MTBH) [8]. Each plate contained standard reactions of 50 nmol, 100 nmol, and 150 nmol of glucose. Change in color was read with a Tecan Sunrise microplate absorbance reader at 620 nm.

### Lignin determination

Lignin content was quantified using the acetyl bromide method according to *Foster et al*. [27]. Briefly, 3mg of biomass alcohol insoluble residue (AIR) were weighed into a 5 mL volumetric flask, and 250 μL of freshly prepared acetyl bromide solution (25% v/v acetyl bromide in glacial acetic acid) was added. Samples were incubated at 50°C for 2h, followed by a further 1h, mixing every 15min. Samples were taken to 5 mL with glacial acetic acid and mixed. The absorption was read using a Shimadzu UV 1800 spectrophotometer (http://www.shimadzu.com) at 280nm. Lignin content was (μg x mg^-1^ cell wall) determined using the following formula: Lignin Content = Absorbance / (Coefficient x Path length) x (Total volume/Biomass weight) x 100. The coefficient used for grasses was 17.75.

### Statistical analyses

All statistical analyses were conducted with the software R version 3.2.3 [28]. Data was adjusted to a linear model with the function *lm* and the significance was established using analysis of the variance (ANOVA) (function *aov*, package agricolae [29]. Differences between species or genotypes were determined by Tuckey HSD test (P ≤ 0.05) (function *LSD*.*test*, agricolae package). Pearson correlations were calculated with *cor* function (*stats* package) and all boxplot and art-graph were depicted with *boxplot* function (*ggplot2* package [30]). The main assumptions of linear model were confirmed using the Shapiro-Wilk test for normal distribution (function *shapiro*.*test*, stats package [28] and by the Levene test for homogeneity of variances (function *leveneTest*, package car [31]) and variables were transformed if required.

## Results and discussion

### Variation of the saccharification potential in a range of cereal cultivars

**Fig 1:**
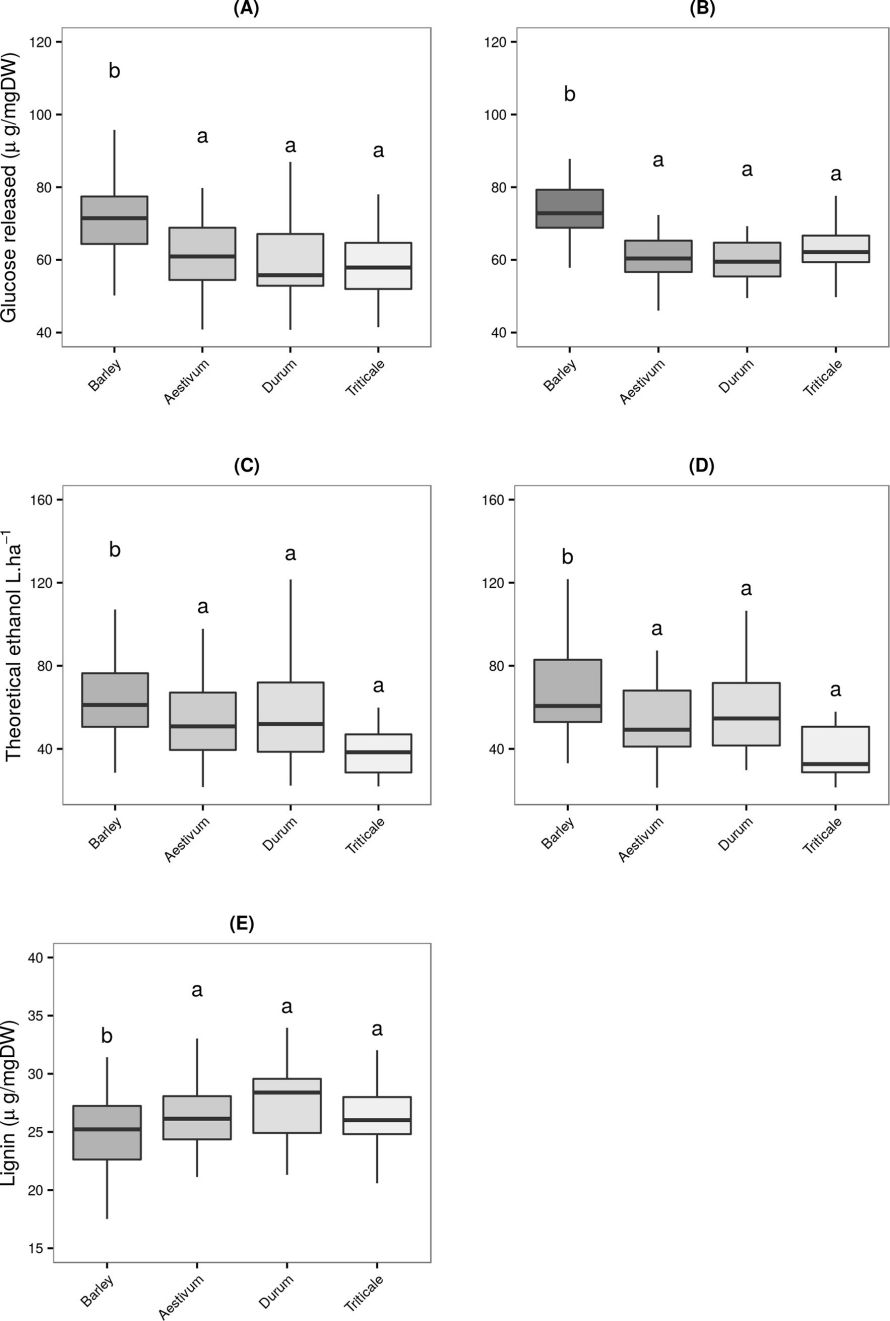
Comparative yield of glucose released in *barley*, *wheat* and *triticale* under different saccharification conditions. Boxplot of glucose’s quantification released for *wheat*, *barley* and *triticale* under different saccharification conditions. (a) IAS; (b) CNAP. Mean (*line*), 25^th^-75^th^ percentile (*box*) and 10^th^-90^th^ percentile (whiskers) of glucose released for each genus. For each saccharification method, bars with different letters are significantly different (ANOVA, Tuckey HSD test, P≤0.05).

To assess the differences in recalcitrance among species and cultivars of triticale, wheat (*T*. *durum* and *T*. *aestivum*) and barley, all samples were analysed for saccharification potential using the IAS and CNAP methods described above. Glucose yields were standardized using inter-plate checks to control inter-plate variance. ANOVA analysis revealed significant differences among species, barley being the species with the highest saccharification potential (Fig 1A).

To validate the results obtained through IAS method, a biological replicate of each sample was analysed using the CNAP method, as control. The correlation of standardized glucose yields between IAS and CNAP methods was moderate (R^2^ = 0.5688) but it is significantly higher than the values reported by Lindedam et al.[16] for two high throughput systems (R^2^ = 0.2139), differences between IAS and CNAP could be due to the different methods used for the quantification of the sugars released. IAS determines only glucose, while CNAP determines all sugars as reducing sugars. Lindedam et al. [16] analysed three different methods, but only reported their best correlation which implies that the other correlations were lower. Both methods used here show that barley presents the highest saccharification potential (Fig 1B). Further analyses were conducted to evaluate the relative recalcitrance among genotypes for each genus/species (Table 2).

**Table 2:** Mean values of total sugar released (μg/mgDW) for sixty-six accessions under IAS-CSIC saccharification conditions. Post-hoc test independently for all genotypes in each. The Study in *Wheat* was made with LSD test (p ≤ 0.05) with Benjamini-Yekutieli p-values adjust. Values with same letter are not significantly different at level 0.05.

*T*.*aestivum* Genotype	Glucose Yield	*T*.*durum* genotype	Glucose yield	*Barley* genotype	Glucose Yield	*Triticale* genotype	Glucose Yield
Caledonia	75.11 a	Avalon	78.4 a	OWB recessive	98.00 a	Juanillo 95	68.32 a
Kanqueen	68.94 ab	NY18/Clark's Cream 40–1	69.55 ab	Steptoe	89.62 ab	Currency	65.5 ab
Excalibur	68.6 abc	IDO444	66.05 abc	Apex	83.24 abc	Yoreme Tehuacan 75	63.38 abc
SS550	67.93 abc	Rugby	63.59 abc	Golden Promise	79.56 abc	Armadillo 130	59.53 abcd
USG3209	67.41 abcd	UC1113 YR36 Gpc-B1	63.52 abc	Capa	76.95 bcd	Drira	58.69 abcd
Avocet	66.94 abcd	Jupateco	58.15 bcd	Lina	75.57 bcd	Rahum	56.48 abcd
Cayuga	66.45 abcd	GRA614A	56.04 bcd	Fredrickson	73.10 bcd	Zebra	55.07 abcd
McNeal	65.37 abcd	Amadina	53.45 bcd	Clipper	73.07 bcd	Navajoa	54.05 bcd
QCB36	64.68 abcd	Weebill_1	53.27 bcd	Azumamugi	72.07 bcd	Wapiti	51.36 cd
P92201	64.58 abcd	CO940610	50.06 cd	Dicktoo	71.60 bcd	Kramer	47.76 d
Renan	63.59 abcd	Rio Blanco	47.1 d	Igri	71.55 bcd		
P91193	63.57 abcd			Mokusekko	69.71 bcd		
CIGM90.248	62.71 abcd			Koa	67.44 bcd		
OS9A	62.33 abcd			OWB dominant	66.49 cd		
Penawawa	59.83 bcd			Stander	65.84 cd		
M6	59.79 bcd			Franka	65.44 cd		
UC1110	57.44 bcd			Morex	65.34 cd		
Thatcher	57.23 bcd			Kanto Nakte Gold	64.88 cd		
Jaypee	56.07 bcd			Vada	64.33 cd		
TAM107 R7	56 bcd			Franklin	62.06 cd		
Anza	55 bcd			L94	52.41 d		
Opata85	53.61 cd						
Bobwhite	51.1 d						
Perico	50.72 d						

**Fig 2:**
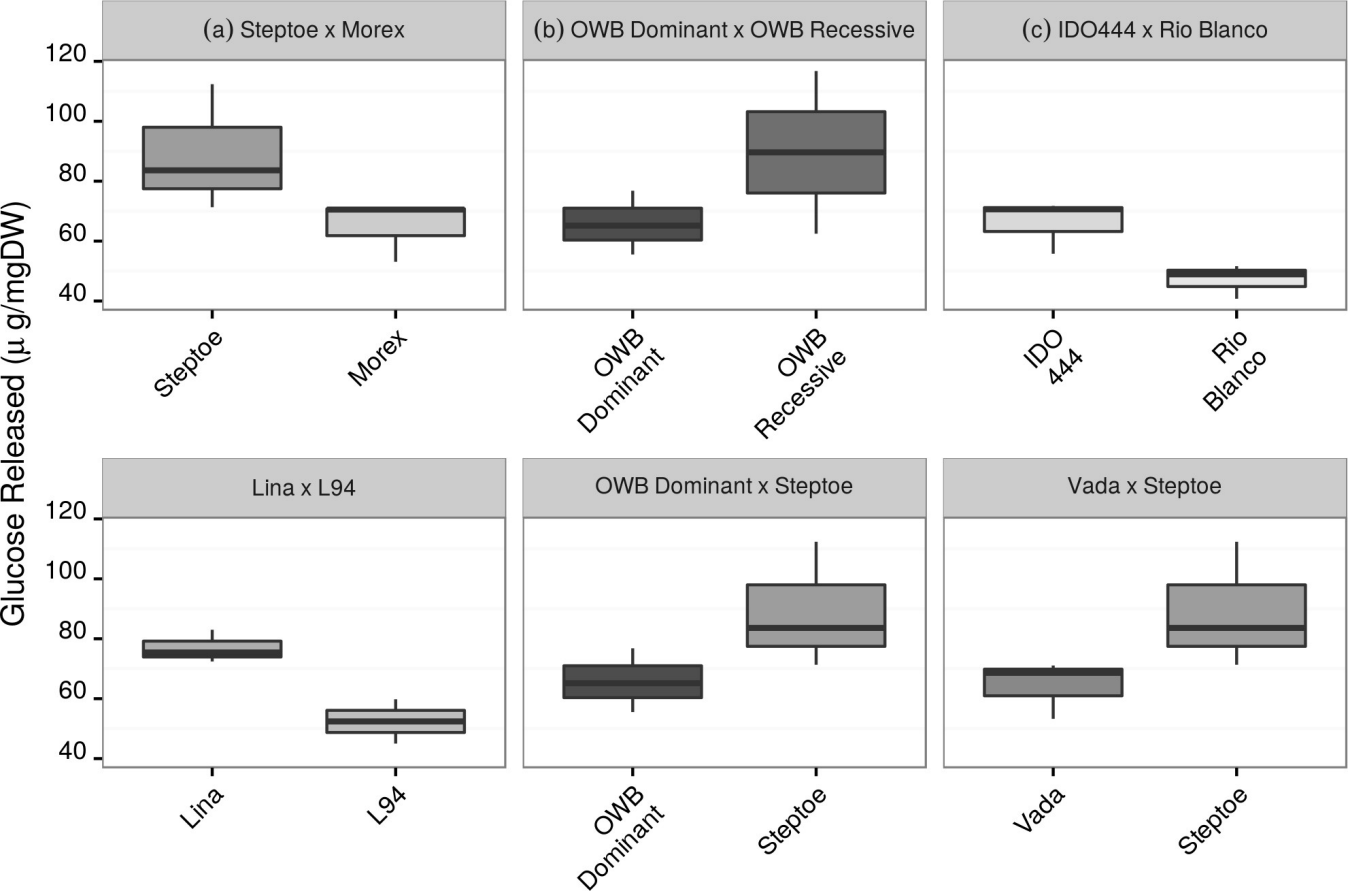
Yield of glucose released in selected *barley* and *wheat* lines. Boxplot of glucose’s quantification. Mean (*line*), 25^th^-75^th^ percentile (*box*) and 10^th^-90^th^ percentile (whiskers) of glucose released for each genotype. Each graph (a to f) shows significant differences at significance level of 0.05 (using IAS-CSIC saccharification conditions) between parental lines used for the development of mapping populations in the literature. Differences shown in graphs a and b were also significant using the CNAP saccharification conditions, and differences shown in graph c was the only one significant different for *wheat*.

Significant differences were detected among barley, wheat and triticale genotypes. In the screening of the 66 cultivars of wheat, barley and triticale, we are able to identify a large variability in the enzymatic hydrolysis of the cell walls of straw. The variability for saccharification among cultivars of different species ranged between 47.09 μg/mg DW and 89.62 μg/mg DW (mean values) using the IAS method, and from 51.59 to 85.07 μg/mg DW using the CNAP method. The variance coefficients (CV) between all genotypes in this trial were 14.7% and 12.2% for the IAS and CNAP methods, respectively. *H*. *Chilense* had a coefficient of variation of 11.6% and 8.54%, *T*. *Aestivum* of 9.6% and 7.6%, *T*. *Durum* of 15.1% and 7.82%, and *Triticosecale* of 9.8% and 6.2%, respectively. The differences between methods for CVs between cultivars of each species are always higher for the IAS method. This could be explained to a large extent because in the IAS method only one 96-well plate could be assayed each time, whereas in the high-throughput method of CNAP a larger number of plates per assay (usually six). In terms of variability in cell wall saccharification, similar results have been previously reported in other collections of different cultivars [11,32,33]. The block factor was also significant in the ANOVA analysis, but it is likely related to a short flooding period during the growing season. A significant block effect was also reported by [19] due to a short period of drought stress. Taken together, these results suggest that the water balance during the crop cycle could marginally affect the release of glucose. In the present work we do not have the possibility of separating the environmental effect of experimental error, but environmental interactions on the degradability of the cell wall have been previously investigated [10,32]. However, several genotypes differing in biomass recalcitrance to enzymatic hydrolysis have been used as parental lines in mapping populations for different traits. These mapping populations constitute a valuable resource for *barley* genetic studies. Indeed, since the development of the Steptoe x Morex and OWB populations [34] they have been successfully used for genetic mapping, including regulatory genes [35] or resistance to leaf stripe [36]. Furthermore, both mapping populations were used to develop a consensus SNP genetic linkage map in *barley* [37]. Given the contrasting saccharification potential of the parental lines, the *barley* mapping populations Steptoe × Morex, Vada × Steptoe, OWB Dominant × Steptoe, OWB Dominant × OWB Recessive, and Lina × L94, could be used to identify the genetic factors underlying differential recalcitrance (Fig 2A). Similarly, the IDO444 × Rio Blanco mapping population [38] could be used in *wheat* (Fig 2C). However, only the OWB populations and Steptoe × Morex should be considered for mapping purposes since the CNAP method did not detect significant differences at *p* < 0.05 between the other parental listed above.

### Determinants of sugar yield

**Fig 3:**
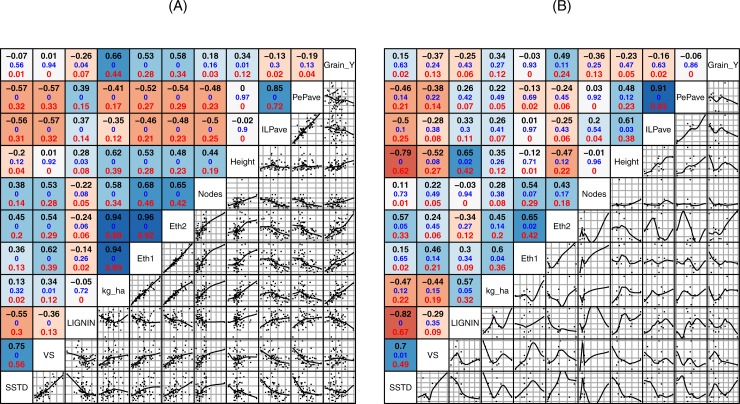
Scatter Plot and Pearson’s correlation coefficient matrix for comparison among phenotyping, saccharification and theoretical ethanol data. Pairwise correlation analyses were performed for all assayed genotypes (a) and the 10 best genotypes for biomass yield (b). The upper panel above the diagonal shows Pearson’s correlation coefficients, p-value and regression coefficient. The lower panel below the diagonal gives their scatter plot. (SSTD = Saccharification standardized values under CNAP conditions, VS = Saccharification standardized values under IAS-CSIC conditions, Kg_ha = estimated weight of straw by hectare, Eth1 = Theoretical ethanol calculated with CNAP’s saccharification values and estimated biomass, Eth2 = Theoretical ethanol calculated with IAS-CSIC’s saccharification values and estimated biomass, ILPave = Average for straw wall thickness for largest internode, PePave = Average for straw wall thickness for peduncle, and Grain_Y = grain yield).

Fig 3 shows the degree of correlation between a number of phenotypic characters and saccharification in all genotypes. Lignin content presents a significant negative correlation with sugar yield (r = -0.55) for all genotypes (Fig 3A), which is in agreement with previous results by Lindedam et al. [39]. When we compared the top 10 genotypes for biomass yield (Fig 3B), we found a stronger Pearson correlation (r = -0.82) and a better relationship between saccharification and lignin content. These results are comparable to previous findings in *Solanum pennellii* by Caruso et al. [40], transgenic alfalfa lines by Chen et al. [41] and *Arabidopsis thaliana* by Van Acker et al [42]. Collectively these results suggest that lignin content should be considered in breeding for saccharification potential. In the current study we observed a negative correlation (r = -0.79) between plant height and saccharification using the CNAP method and a positive correlation between plant height and lignin content (r = 0.65) (Fig 3B), both correlations for high biomass yield selected lines. This relationship between plant height and plant cell wall recalcitrance could be due to the requirement of increased lignin for mechanical stiffness with the consequent reduction in saccharification. Similar results were showed by [11] and [43]. The negative correlation between plant height and degradability could also partly be explained by higher plants having relatively smaller leaf fraction. For correlation analysis with all samples, we could not see correlation between height and degradability; this fact could be explained because breeding programs for semi-dwarf cultivars may in fact have affected the degradability of modern cultivar [44]. ILPave (Average for straw wall thickness for largest internode) and PePave (Average for straw wall thickness for peduncle) showed a significant negative correlation with degradability, theoretical ethanol and number of nodes, and also showed a positive correlation between thickness and lignin content (Fig 3A). Generally, *barley* has a higher number of nodes and low wall thickness, which is consistent with high saccharification and low lignin content results showed in Fig 1. Differences in lignin content in cell wall of one genotype of wheat, one barley, and one triticale straw, have been reported previously, showing that barley contains less lignin than wheat [45,46]. Our results obtained from many genotypes for each species are in agreement with previous reports and extend the observation across genotypes. Plants with the same height and stems with low wall thickness will have more short internodes, implying more numbers of nodes, and consequently are less susceptible to lodging. Correlations between lodging resistance, thickness and number of nodes were shown by Jezowski et al. [47], Tandon et al. [48], and Brady et al. [49] On the other hand, as reported Saint Pierre et al. [50] thickness is an ideal factor to maximize water soluble carbohydrate reserves, and it appears to be important under water limited conditions, where these could be mobilized for grain filling. ILPave and PePave were uncorrelated with plant height and grain yield, hence allowing breeding for that character without compromising high grain yield. Moreover, we could assess that grain yield and saccharification are not correlated (Fig 3A), establishing a degree of independence between these two traits.

#### Final remarks

In the current work we analysed a collection of wheat (*T*. *durum* and *T*. *aestivum*), barley and triticale genotypes in order to investigate interspecific and intraspecific differences. The methodology adapted at IAS could be useful for genotype selection in biomass quality since it shows a good degree of concordance with previous methodologies. Thus, it would be useful for the identification of improved varieties with good saccharification potential in a breeding program. Collectively, our results indicate that barley is a better source of lignocellulosic material for bioethanol production than wheat and triticale. The ranking of genotypes was slightly different with IAS and CNAP methods, but the most contrasting genotypes were picked up by both methodologies. Interestingly, some of the most dissimilar genotypes have been used to develop mapping populations in barley. For instance, both Steptoe × Morex and OWB Dominant × OWB Recessive barley mapping populations would be good tools for the identification of the genetic basis of saccharification-related traits. Finally, correlation analyses suggest that sugars released, lignin content and its correlation with straw wall thickness would be good predictors of biomass degradability in breeding programs. Furthermore, the lack of correlation between grain yield and saccharification suggests that it would be possible to select genotypes with low recalcitrance and high grain yield for dual use (grain and energy).

## Supporting information

S1 Fig Enzyme optimization. Glucose released in wheat genotypes (Anza, Bobwhite and Perico) with different concentrations of enzyme cocktail. R^2^ values correspond to different wheat genotypes and enzyme concentrations between 2 and 0.0078 μL/mg DW.
(TIFF)
