# Supplementary figures and images for: Biomass recalcitrance in barley, wheat and triticale straw: Correlation of biomass quality with classic agronomical traits
Source: PLoS One. 2018 Nov 7;13(11):e0205880. doi: 10.1371/journal.pone.0205880 (PMC6221549; doi:10.1371/journal.pone.0205880)

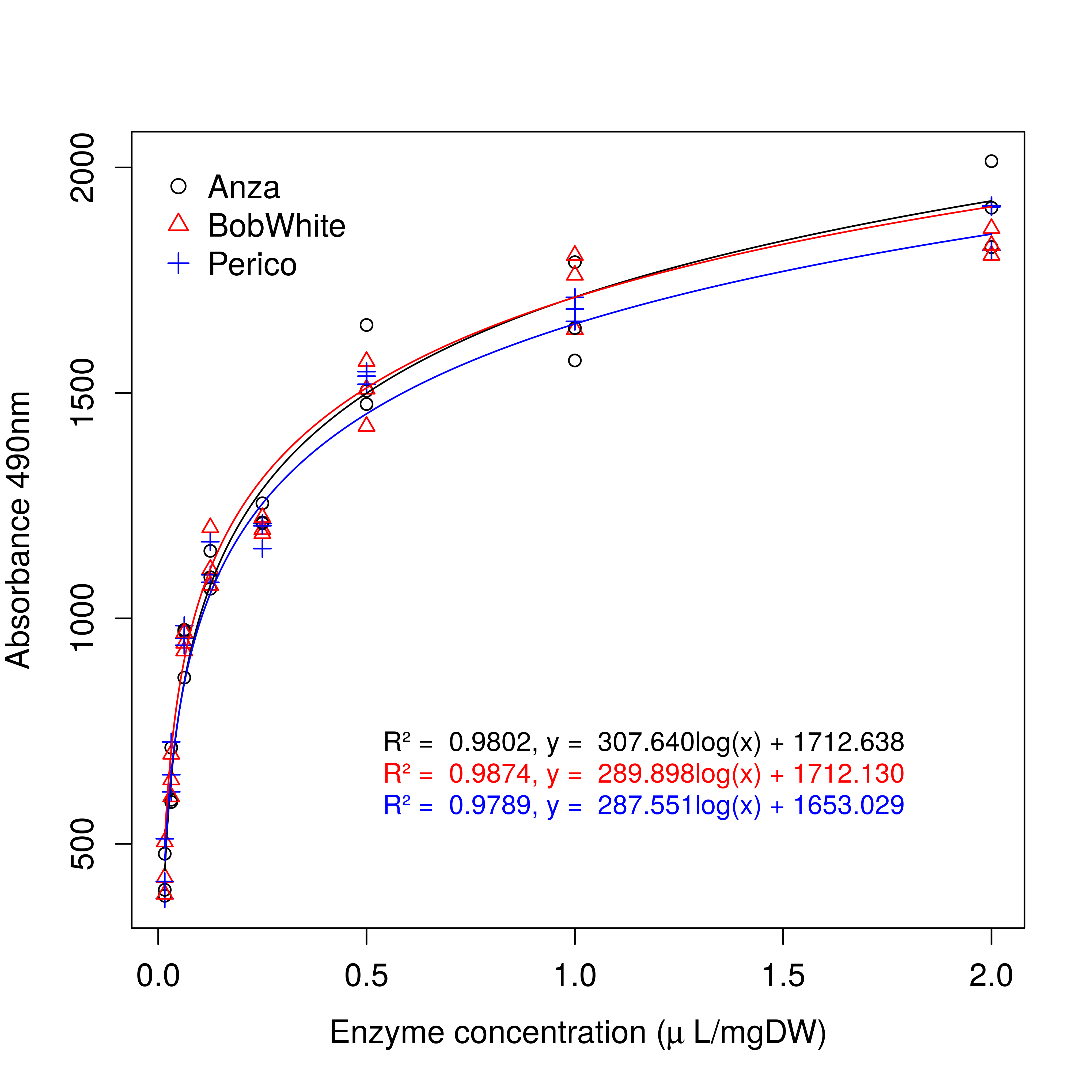

Supplement: S1 Fig — Glucose released in wheat genotypes (Anza, Bobwhite and Perico) with different concentrations of enzyme cocktail. R2 values correspond to different wheat genotypes and enzyme concentrations between 2 and 0.0078 μL/mg DW. (TIFF) [file pone.0205880.s001.tiff]
